# Supplementary material for: Computational redesign of the Escherichia coli ribose-binding protein ligand binding pocket for 1,3-cyclohexanediol and cyclohexanol
Source: Sci Rep. 2019 Nov 15;9:16940. doi: 10.1038/s41598-019-53507-5 (PMC6858440; doi:10.1038/s41598-019-53507-5)
Supplement: Supplementary file 1 — Supplementary information [file 41598_2019_53507_MOESM1_ESM.pdf]

Supplementary Information to

Computational redesign of the *Escherichia coli* ribose–binding protein ligand binding pocket for 1,3-cyclohexanediol and cyclohexanol

Diogo Tavares*, Artur Reimer*[#], Shantanu Roy*, Aurélie Joublin, Vladimir Sentchilo and Jan Roelof van der Meer†

Department of Fundamental Microbiology, University of Lausanne, 1015 Lausanne, Switzerland

† To whom correspondence should be addressed:

J. R. van der Meer, Department of Fundamental Microbiology, Bâtiment Biophore, Quartier UNIL-Sorge, University of Lausanne, 1015 Switzerland; janroelof.vandermeer@unil.ch; Tel: +41 21 692 5630.

Table S1- Composition of mineral medium (MM) and low phosphate mineral medium (MM LP) used in this study.

Component	Mineral medium	Low phosphate mineral medium
Na_2HPO_4	60 g	0.36 g
KH_2PO_4	30 g	0.33g
NaCl	5 g	
NH_4Cl	10 g	

Recipe for 1L. pH set to 7.4
Supplemented with 1 ml l^{-1} of Hutner's trace mineral solution as per reference (1) for 21C medium.

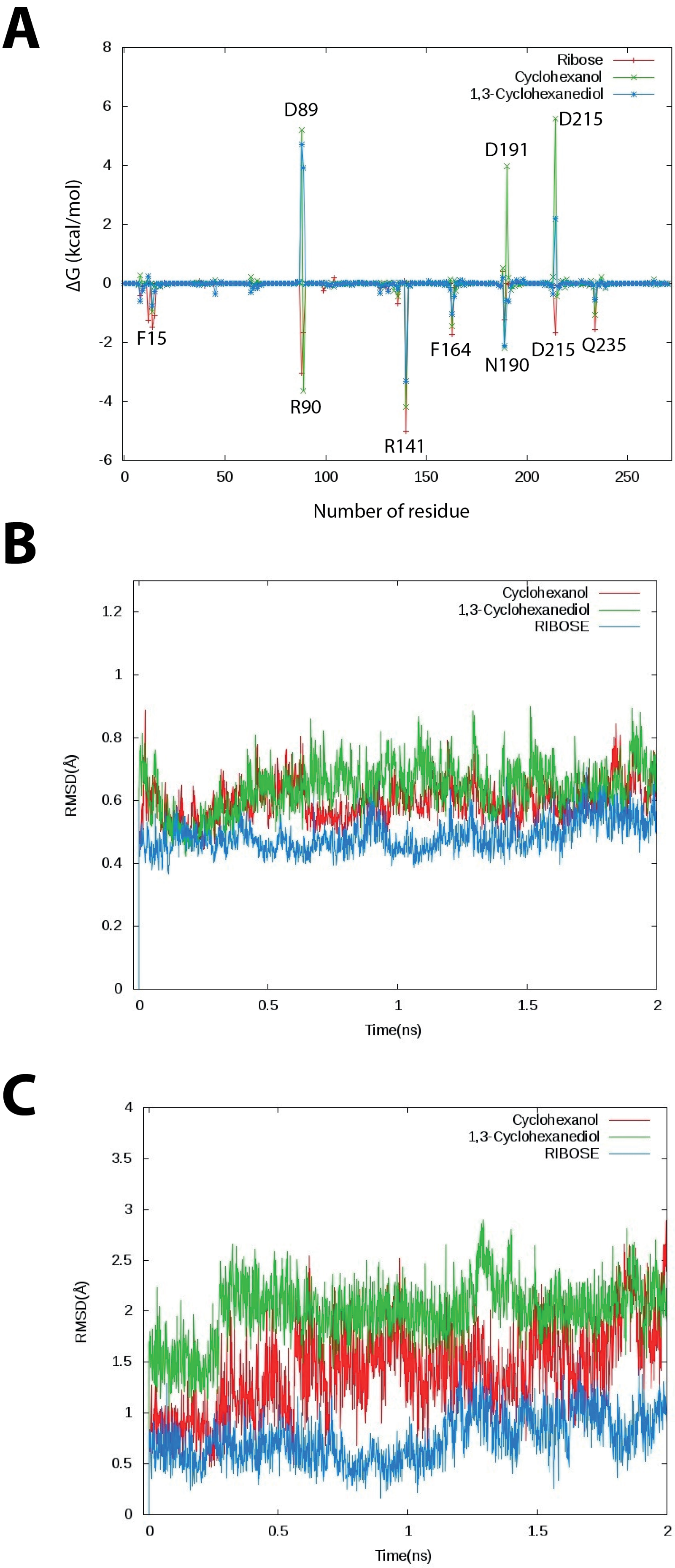
A
Ribose
Cyclohexanol
1,3-Cyclohexanediol
ΔG (kcal/mol)
Number of residue
D89
D191
D215
F15
R90
R141
F164
N190
D215
Q235
8
6
4
2
0
-2
-4
-6
0
50
100
150
200
250
B
Cyclohexanol
1,3-Cyclohexanediol
RIBOSE
RMSD(Å)
Time(ns)
1.2
1
0.8
0.6
0.4
0.2
0
0
0.5
1
1.5
2
C
Cyclohexanol
1,3-Cyclohexanediol
RIBOSE
RMSD(Å)
Time(ns)
4
3.5
3
2.5
2
1.5
1
0.5
0
0
0.5
1
1.5
2

Figure S1- Interactions between RbsB wild-type and the ligands ribose, 13CHD or CH using docking and molecular dynamics simulations. A) Contribution of each residue of RbsB to the change of Gibbs free energy ΔG (kcal/mol) during binding of the indicated ligand molecules using per-residue binding free energy decomposition based on Molecular Mechanics-Generalized Born Surface Area (MM-GBSA) method. B) Average spatial deviation of the RbsB binding pocket with placed ligands (ribose, 13CHD or CH) during 2 ns as root-mean squared deviation. C) As B, but taken from the ligand positions.

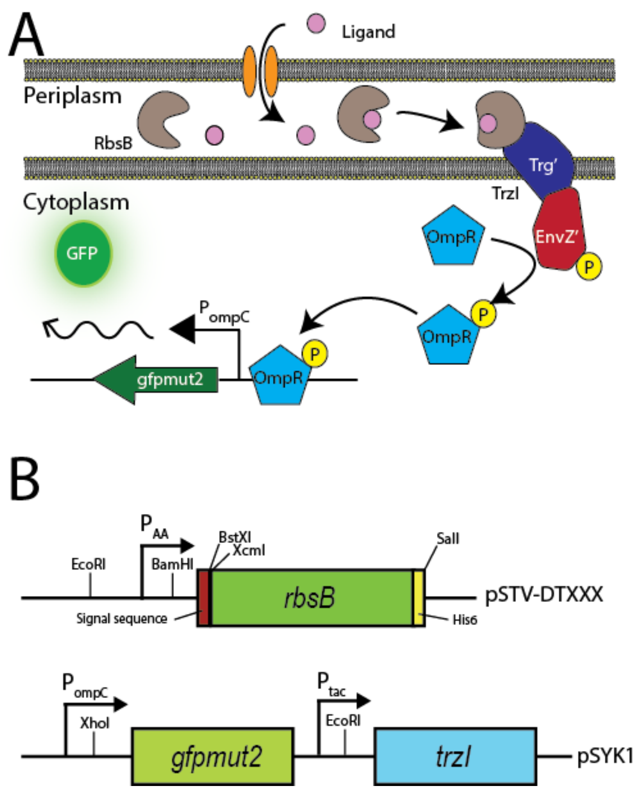

Figure S2 - The hybrid RbsB-OmpR signaling chain of the *E. coli* Trz1-OmpR bioreporter strain. (A) Ribose (ligand) is bound by the ribose-binding protein (RbsB), which docks to the Trz1 hybrid receptor (fusion between the periplasmic part of Trg and the cytoplasmic part of EnvZ). The binding starts a phosphorylation cascade, where OmpR is phosphorylated and increases transcription of gfp from the ompC promotor. (B) Scheme of relevant plasmids used in this work. Plasmid pSTV-DTXXX expresses the *rbsB* or mutant *rbsB* gene with its translocation signal sequence and hexahistidine tag (His_6) under control of the weak constitutive P_{AA} promoter (2). Plasmid pSYK1 contains the *gfpmut2* gene under the *ompC* promoter control and the *trzI* gene under control of P_{tac}. Relevant restriction sites are indicated.

Supplementary References

1. **Gerhardt P, Murray RGE, Costilow RN, Nester EW, Wood WA, Krieg NR, Phillips GB (ed).** 1981. Manual of methods for general bacteriology. American Society for Microbiology, Washington, D.C.
2. **Alper H, Fischer C, Nevoigt E, Stephanopoulos G.** 2005. Tuning genetic control through promoter engineering. Proc Natl Acad Sci U S A **102:**12678-12683.
